# Identification of a Subtype of Hepatocellular Carcinoma with Poor Prognosis Based on Expression of Genes within the Glucose Metabolic Pathway

**DOI:** 10.3390/cancers11122023

**Published:** 2019-12-14

**Authors:** Xiaoli Zhang, Jin Li, Kalpana Ghoshal, Soledad Fernandez, Lang Li

**Affiliations:** 1 Department of Biomedical Informatics, The Ohio State University, Columbus, OH 43201, USA; jin.li@osumc.edu (J.L.); Soledad.Fernandez@osumc.edu (S.F.); Lang.Li@osumc.edu (L.L.); 2 Department of Pathology, College of Medicine, The Ohio State University, Columbus, OH 43201, USA; Kalpana.Ghoshal@osumc.edu

**Keywords:** hepatocellular carcinoma, glucose metabolism, gene expression, clinical outcomes, clustering, stemness, down-regulated metabolism

## Abstract

Hepatocellular carcinoma (HCC) is the most prevalent primary cancer and a highly aggressive liver malignancy. Liver cancer cells reprogram their metabolism to meet their needs for rapid proliferation and tumor growth. In the present study, we investigated the alterations in the expression of the genes involved in glucose metabolic pathways as well as their association with the clinical stage and survival of HCC patients. We found that the expressions of around 30% of genes involved in the glucose metabolic pathway are consistently dysregulated with a predominant down-regulation in HCC tumors. Moreover, the differentially expressed genes are associated with an advanced clinical stage and a poor prognosis. More importantly, unsupervised clustering analysis with the differentially expressed genes that were also associated with overall survival (OS) revealed a subgroup of patients with a worse prognosis including reduced OS, disease specific survival, and recurrence-free survival. This aggressive subtype had significantly increased expression of stemness-related genes and down-regulated metabolic genes, as well as increased immune infiltrates that contribute to a poor prognosis. Collectively, this integrative study indicates that expressions of the glucose metabolic genes could be used as potential prognostic markers and/or therapeutic targets, which might be helpful in developing precise treatment for patients with HCC.

## 1. Introduction

Hepatocellular carcinoma (HCC) is the most prevalent primary cancer worldwide and the third leading cause of cancer-related mortality [1,2]. It is highly malignant, recurrent, resistant to drugs, and typically diagnosed at a late stage [3,4]. HCC poses a global health challenge due to the rising incidence and low survival rate, especially in the developing world [5,6]. The high clinical heterogeneity of this disease and lack of a molecular classification hampered the development of effective treatment standards. Therefore, there is urgent need to develop specific molecular markers that contribute to HCC progression and can be used as diagnostic, prognostic, or therapeutic targets.

Energy metabolism, particularly glucose metabolism, is the most affected process during the transition of normal cells to cancer cells [7]. Reprogramming of glucose metabolism has been recognized as a key hallmark of cancer cells. This alteration in glucose metabolism accompanied by enhanced uptake of glucose and conversion of pyruvate to lactate, known as the Warburg effect, is to satisfy the increasing requirement of macromolecular synthesis to maintain unregulated rapid cellular proliferation and survival [8,9]. Interestingly, the augmented uptake and metabolism of glucose generally correlate with a poor prognosis of many tumor types, including HCC [10,11,12]. The landmark TCGA (The Cancer Genome Atlas) analysis of HCC highlighted a key role of metabolic reprogramming during HCC progression with down-regulated metabolic genes such as ALB (albumin), APOB (apolipoprotein B), and CPS1 (carbamoyl phosphate synthetase I) [13]. Thus, identification of consistently deregulated metabolic genes or pathways in HCC, especially glucose metabolic related pathways, will accelerate future mechanistic studies in identifying diagnostic, prognostic, or therapeutic targets for HCC.

**Figure 1:**
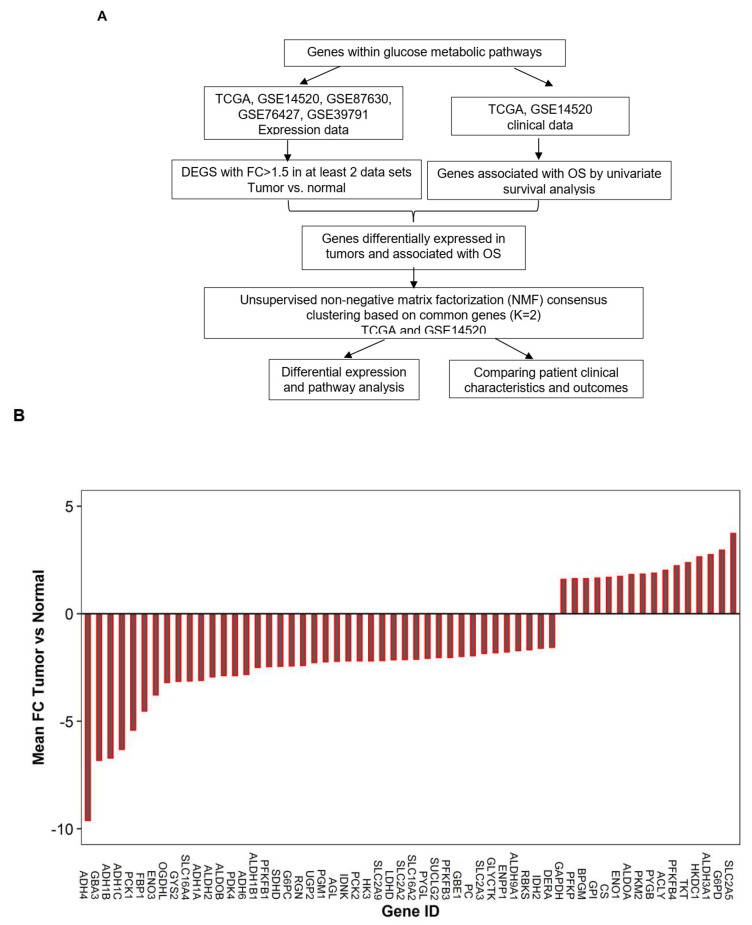
Outline of the planned work for this study (**A**) and waterfall plot showing the deregulated genes within the glucose metabolic pathway in Hepatocellular carcinoma (HCC) (**B**). The gene expression was first compared between the tumor and the normal cells as described in the methods (FC ≥ 1.5 and *p* < 0.0056), and then the genes that were significantly differentially expressed in at least two of the five data sets were chosen. The fold change on the Y-axis was the mean fold change of the gene expression comparing the tumor to the normal cells averaged across the number of data sets in which it showed a differential expression. OS: overall survival. FC: Fold change.

Besides upregulation in glycolysis, tumor cells also exhibit increased glycogen metabolism [14], and gluconeogenesis [15] as opposed to normal cells. In order to understand dysregulation of the glucose metabolism pathway in tumor cells, it needs to include genes involved in glycolysis, Tricarboxylic Acid Cycle (TCA), and the pentose phosphate pathway (PPP), which breaks down glucose to provide metabolites for necessary energy production. On the other hand, it also needs to include genes involved in gluconeogenesis, glycogenolysis, and glycogenesis pathways that can balance the glucose level in blood, as well as genes regulating these processes. Therefore, in the present study as illustrated in Figure 1A, we focused on identifying genes that were consistently dysregulated within the glucose-related metabolic pathways and investigating their predictive power for patient outcomes. Moreover, based on the dysregulated glucose metabolic genes, we could identify distinct molecular subtypes that were significantly associated with the prognostic outcomes of HCC.

## 2. Results

### 2.1. Genes within Glucose Metabolic Pathway Are Dysregulated in Human HCC Tumor Cells

To understand the alteration of glucose metabolic pathway in clinical HCC samples, genes involved in the pathways of glycolysis, pentose phosphate, Tricarboxylic Acid Cycle (TCA) cycle, gluconeogenesis, and glycogen metabolism were curated from the KEGG (Kyoto Encyclopedia of Genes and Genomes) pathway. In addition, glucose transporter genes, lactate transporter genes, and genes regulating glucose and glycogen metabolism were also included (Table S4). In total, 179 genes involved in glucose metabolism were assessed in primary HCC patient cohorts (Table S4). Five data sets including liver cancer (LIHC) TCGA, GSE14520, GSE89670, GSE39791, and GSE76457 were used for the assessment (Figure 1A and Table S1). By comparing gene expression in primary tumors to adjacent normal tissues or to non-tumor liver tissues for each data separately, 58 genes out of 179 (~35%) were found to be consistently deregulated in at least two data sets with at least a 1.5-fold change (FC) (FDR < 0.05). Among them, 19, 8, 13, and 18 genes were consistently deregulated in all 5, or in 4, 3, and 2 data sets, respectively. Surprisingly, more than 70% (42/58) of the deregulated genes were significantly down-regulated in HCC tumors compared to non-tumor liver tissues (Figure 1B and Table S5).

We observed that genes within the glycolysis pathway were mainly upregulated, including HKDC1, GPI, PFKP, ALDOA, BPGM, GAPDH, ENO1, and PKM2, even though HK3, ALDOB, ENO3, PFKFB1, and PFKFB3 were down-regulated (Figure 1B and Figure 4). However, the genes involved in glycogen metabolism (ENPP1, GBE1, GYS2, UGP2, GBA3, AGL, and PYGL), gluconeogenesis (FBP1, G6PC, PC, PCK1, and PCK2), and TCA cycle (IDH2, SUCLG2, SDHD, and OGDHL) were mainly down-regulated (Figure 1B). There is a mixture of both consistently up-regulated (G6PD and TKT) and down-regulated genes (RBKS, IDNK, GLYCTK, RGN, and DERA) in the pentose phosphate pathway (PPP). For the glucose transporters, except GLUT5 that was upregulated, GLUT2, GLUT3, and GLUT9 were all down-regulated in tumors. In addition, the differentially expressed enzymes involved in pyruvate metabolism (PDK4 and LDHD) and the lactate transporters (MCT3 and MCT8) were all down-regulated as well.

### 2.2. Dysregulated Genes within Glucose Metabolic Pathway Are Associated with HCC Patient Tumor Stage and Survival

**Figure 2:**
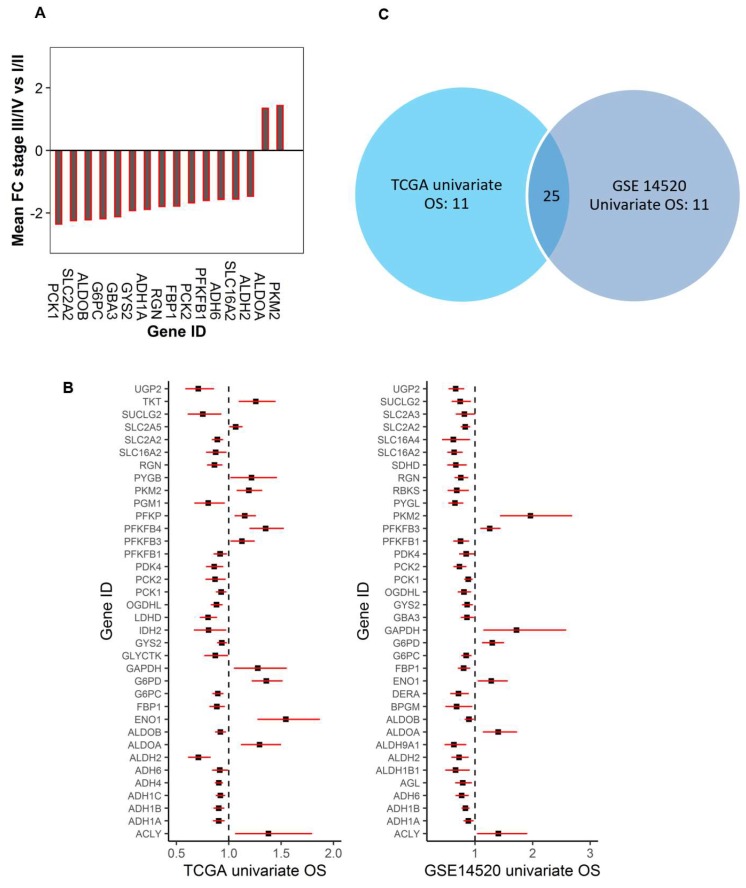
Dysregulated genes within the glucose metabolic pathway associated with the AJCC (American Joint Committee on Cancer) tumor stage and the OS of HCC patients. (**A**). The waterfall plot to show the differentially expressed genes in the tumor were also associated with an aggressive stage of the tumor (*p* < 0.0001). The down-regulated genes were further down-regulated, and the up-regulated genes were further up-regulated in late stage tumors. (**B**). The association of glucose metabolic gene expression with patients’ overall survival in HCC (Left: TCGA, and right: GSE14520) (*p* < 0.05). The forest plots with the hazard ratios (HR) and 95% confidence intervals (CI) showing a survival advantage (HR < 1) or disadvantage (HR > 1) with increased expression of the dysregulated glucose metabolic genes. Univariate Cox proportional hazard regression models were used for the association tests. (**C**). The Venn diagram to show the number of genes that were specific or common for the two data sets based on univariate OS analysis in Fig2B.

We further investigated whether the dysregulated genes in HCC tumors were associated with tumor progression using AJCC (American Joint Committee on Cancer) clinical stage. The tumor stage was dichotomized into an early (I,II) and late stage (III,IV). By comparing the gene expression between the early and late stage using both TCGA and GSE14520 data, we found that 16 dysregulated glucose metabolic genes were consistently associated with the tumor stage in both data sets (*p* < 0.0067, FC > 1.35) (Figure 2A). Of these, PKM2 and ALDOA had further increased expression, while the rest of the 14 genes were further down-regulated in late stage tumors compared to early stage tumors. These genes are involved in glycolysis (ALDOA, ALDOB, PKM2, PFKFB1), glycogen metabolism (GBA3, GYS2), gluconeogenesis (FBP1, G6PC, PCK1, PCK2), and pentose phosphate pathway (RGN). In addition, expression of alcohol dehydrogenase 1A and 6 (ADH1A and ADH6) as well as lactate transporter (SLC16A2: MCT8) were also significantly decreased in late stage tumors (Figure 2A).

To investigate whether the genes consistently dysregulated in HCC tumors could predict patient outcomes, the association between the expression of those genes and overall survival (OS), recurrence-free survival (RFS), disease-free survival (DFS), progression-free interval (PFI), disease-free interval (DFI), and disease-specific survival (DSS) were tested using TCGA and GSE14520. Univariate or multivariate Cox proportional regression models were used for testing. We found that, among the 58 differentially expressed genes, 47 of them were associated with OS in at least one of the two data sets (Figure 2B,C and Table S5) with 36 significantly associated with OS in TCGA and 36 in GSE14520 based on univariate analysis. Among them, 25 genes were significantly associated with OS in both data sets (Figure 2B,C). The rest of the 11 dysregulated genes were not significantly associated with OS even though some of them (ENO3: enolase 3 and PC: pyruvate carboxylase) were differentially expressed in at least four of the five data sets (Figure 1B and Table S5). For the following clustering analysis, only genes that were differentially expressed in the tumor and were also associated with OS by univariate analysis were considered.

**Figure 3:**
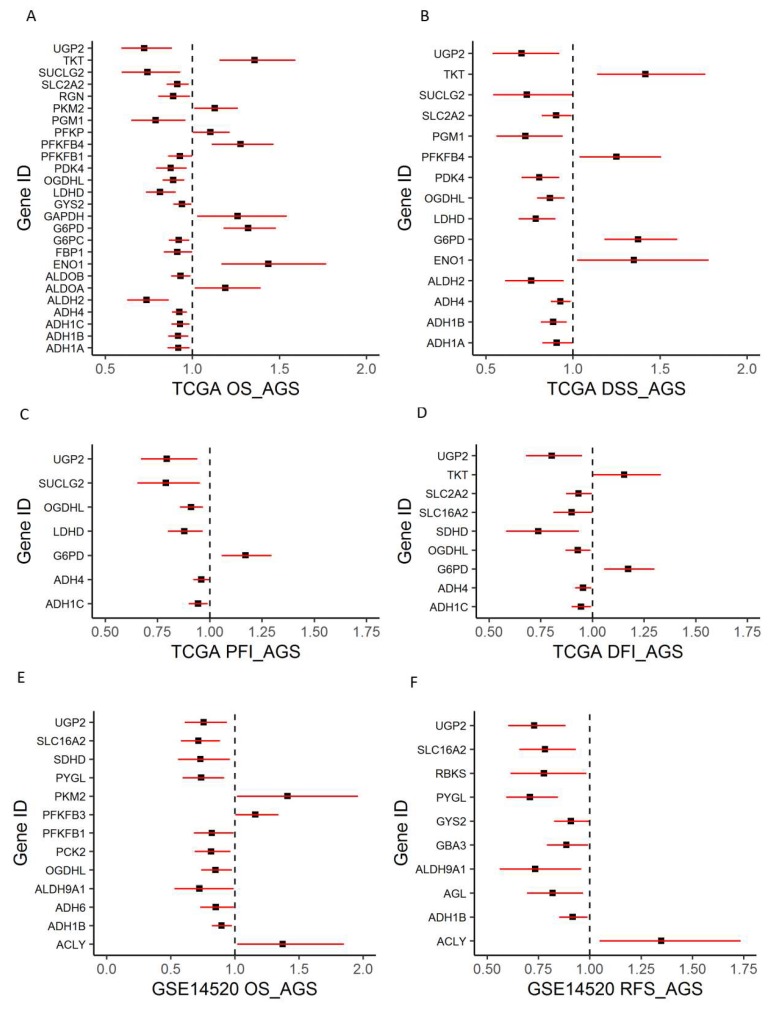
Forest plots to show an association between glucose metabolic gene expression and patient survival outcomes. (**A–D**). Overall survival (OS), disease-specific survival (DSS), disease-free interval (DFI), and progression-free interval (PFI) for TCGA data. (**E–F**) OS and recurrence-free survival (RFS) for GSE14520 data. Multivariate Cox proportional regression models were used for testing while controlling for age, gender, and the tumor stage (AGS) in the model besides gene expression (*p* < 0.05).

Since the tumor stage is a well-known predictor for survival, multivariate analysis controlling for age, gender, and stage in the TCGA data set showed that 26 genes were independent predictors for OS among the 36 genes that were significant for univariate OS (Figure 3A). In addition, 15, 9, and 7 genes were found to be independent predictors for DSS, DFI, and PFI, respectively, among the 58 DEGS (Figure 3B–D) using TCGA data. Among them, UGP2, OGDHL, G6PD, and ADH4 were independent predictors for all four types of survival. For data GSE14520, 13 among the 36 genes were found to be independent predictors for OS after controlling for age, gender, and tumor stage (Figure 3E). Similarly, 10 genes among the 58 DEGS were found to be independent predictors for RFS using data GSE14520 (Figure 3F). Six genes, including UGP2, SLC16A2, PYGL, ALDH9A1, ADH1B, and ACLY, were independent predictors for both OS and RFS for GSE14520 data. By comparing the results from the survival analysis of the two data sets, we found that ADH1B, OGDHL, PFKFB1, PKM2, and UGP2 were significant predictors for OS from both data sets. Notably, all the genes associated with the tumor stage except GBA3 and PCK1 were independent predictors for OS in at least one of the two data sets (Figure 2A and Figure 3A,E).

**Figure 4:**
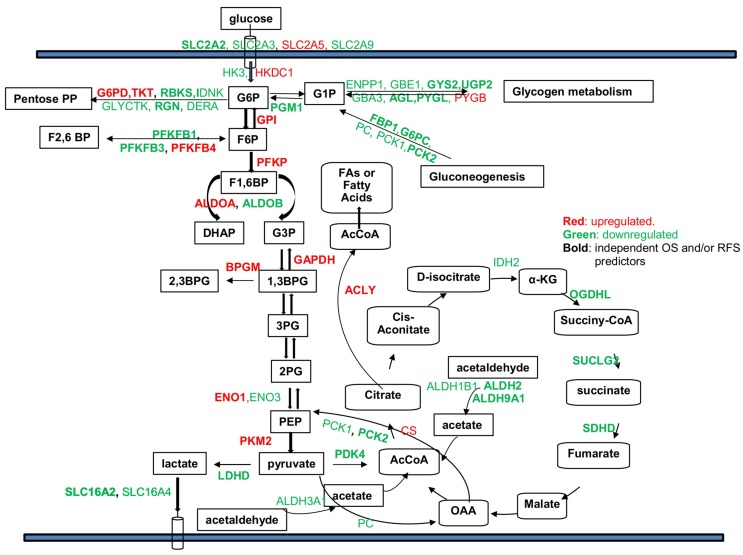
Summary of deregulated genes within different glucose metabolism related pathways and the potential use of certain genes as independent predictors for OS and /or RFS. Red: genes significantly up-regulated in tumors. Green: genes significantly down-regulated in tumors compared to normal tissues. Bold: genes that could be used as independent predictors for OS and /or RFS after controlling for age, gender, and tumor stage in the multivariate Cox regression model (*p*-values < 0.05). ADH1A, ADH1B, and ADH6 were all down-regulated in HCC tumors in all five data sets, but they were not labeled in the graph since their specific function in glucose metabolic pathways is not clear.

Therefore, those genes could be important prognostic markers and/or therapeutic predictors of HCC. Figure 4 summarizes the results showing that the dysregulated genes and their association with OS and/or RFS as independent predictors. Specifically, among the DEGs that are independent survival predictors, we found that UGP2 (uridine diphosphate (UDP)-glucose pyrophosphorylase 2) had significantly decreased expression in all five data sets, and it was also a significant prognostic factor for all the tested patient outcomes. With the decrease of UGP2 expression, there was increased risk for OS, DFS, PFI, DSS, and DFI for HCC patients. In contrast, previous studies in HCC cell lines showed that upregulation of UGP2 was associated with increased metastatic potential, and promoted cell migration and invasion [16]. Therefore, further studies are needed to confirm the role of UGP2 in primary HCC.

### 2.3. Molecular Subtypes of HCCs Based upon Genes Differentially Expressed in Tumors and Associated with Patient OS

**Figure 5:**
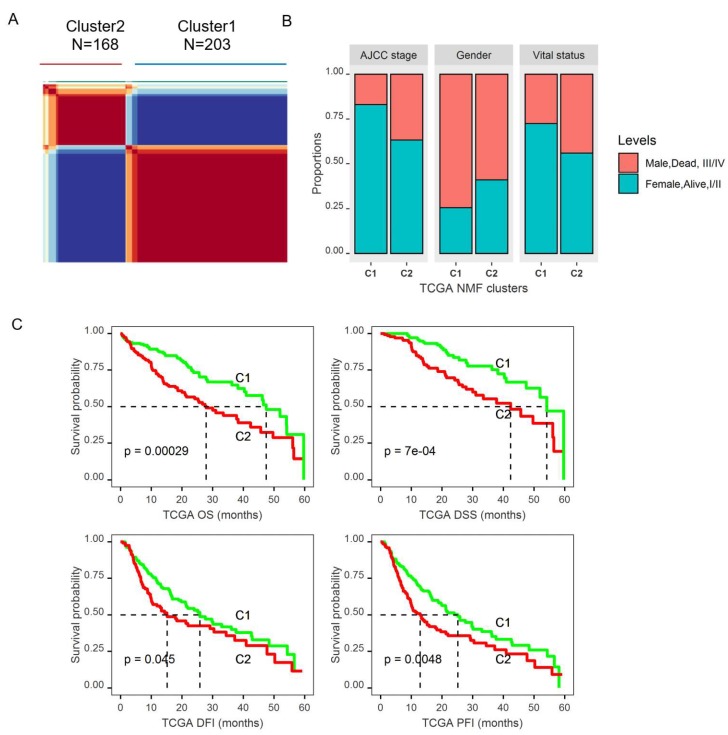
Identification of molecular subtypes of HCC using TCGA data based on the DEGS in the tumor that were also associated with OS from the univariate analysis. (**A**). The non-negative factorization (NMF) consensus plot for TCGA identified two distinct HCC subtypes: Cluster1 and Cluster2. (**B**). Histogram showing that Cluster2 (C2) patients had a higher proportion of female patients, more patients with a late stage tumor, and more death events compared to Cluster1(C1). (**C**). Log-rank tests showed that Cluster2 patients had significantly worse five-year OS, DSS, DFI, and PFI (*p* < 0.05).

From the heatmap of TCGA data based on the 47 differentially expressed glucose metabolic genes that were also associated with OS (Figure S1), we noticed that the patient samples were separated into two clusters, with one being in the same cluster as the adjacent normal samples, while the other cluster was independent. Therefore, we further evaluated whether those 47 genes could predict prognostic subgroups. We applied a non-negative matrix factorization (NMF) consensus cluster analysis using TCGA data based on 47 genes that yielded two distinct clusters (Figure 5A and Figure S2A). Cluster2 patients were found to be significantly younger (mean age 57.1 vs. 61.3 years old, *p* = 0.0029), with more women (*p* = 0.0016), and a higher proportion of patients with higher grade (III,IV) tumors (*p* < 0.0001) (Table S2 and Figure 5B). More importantly, Cluster2 patients were found to have a significantly worse five-year overall survival than Cluster1 patients, with a median survival of 27.9 months (95% CI: 20.9, 38.3) for Cluster2 vs. 47.43 months (95% CI: 40.33, 54.13) for Cluster1 (*p* = 0.0003) (Table S2 and Figure 5C). Those patients also had significantly worse PFI (*p* = 0.006), DFI (*p* = 0.042), and DSS (*p* = 0.0009) based on five-year survival analysis (Table S2 and Figure 5C). The five-year OS analysis including the stage showed that patients in Cluster2 had significantly worse survival compared to Cluster1 patients within both the early stage and the late stage. In addition, patients with stage III/IV disease had worse DSS in Cluster2 than in Cluster1 (Figure S3A), but no significant survival difference between the two clusters for patients with early stage. After controlling for age, gender, and tumor grade in multivariate Cox proportional hazard regression models, Cluster2 patients still had significantly worse OS (*p* = 0.005, HR: 1.743 with 95% CI (1.182, 2.569)) and DSS (*p* = 0.02, HR: 1.831 with 95% CI (1.093, 3.066)) than Cluster1 patients.

**Figure 6:**
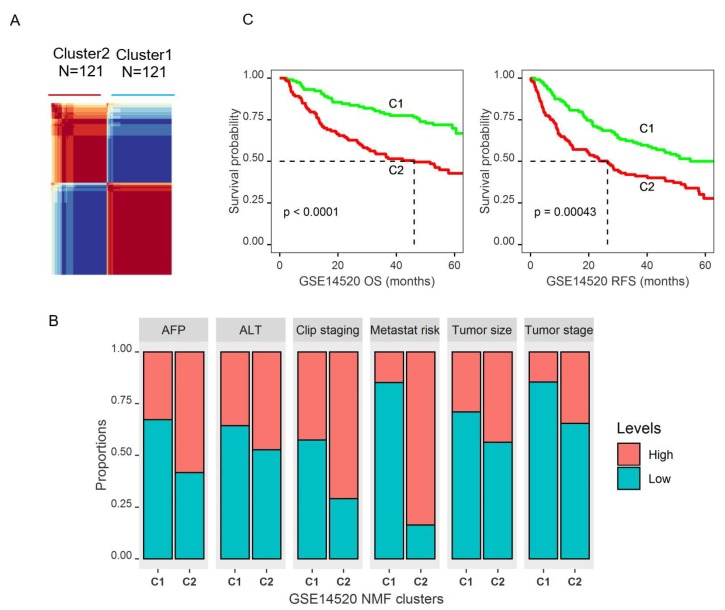
Identification of molecular subtypes of HCC based on the DEGs in the tumor that were also associated with OS using an independent data set GSE14520. (**A**). NMF plot of GSE14520 identified two distinct HCC clusters. (**B**). Histogram to show that Cluster2 patients of GSE14520 had a significantly higher level of AFP expression, bigger tumor size, higher tumor stage, higher clipping stages, and higher predicted risk for metastasis (*p*-values < 0.005). The ALT level was higher in Cluster2 but the difference was not significant. (**C**). Kaplan-Meier survival curves showing that Cluster2 patients had significantly worse five-year OS and RFS (*p* < 0.0045).

To validate the findings that DEGs within glucose metabolic pathways could predict prognostic subtypes, the same method with NMF clustering was applied to data GSE14520. Interestingly, NMF clustering with this data also yielded two distinct clusters (Figure 6A and Figure S2B). Patients in Cluster2 were younger (mean age 49.39 vs. 52.30 years old, *p* = 0.037), had a higher predicted risk for metastasis (84.3% vs. 15.7%, *p* < 0.0001), higher AFP level (*p* < 0.0001), bigger tumor size (*p* = 0.019), and higher proportion of patients with a higher tumor grade (Figure 6B and Table S3). Most importantly, Cluster2 patients also had significantly worse OS (*p* < 0.0001, median survival: not achieved for Cluster1 vs. 46.1 months for Cluster2) and RFS (*p* = 0.0004, median survival: not achieved for Cluster1 vs. 26.4 months for Cluster2) (Figure 6C and Table S3). Kaplan-Meier plots including the tumor stage showed a significant difference of OS and RFS between the two clusters for both early and late stages (Figure S3B). After controlling for age, gender, AFP, tumor size, and tumor stage in multivariate Cox proportional regression model, Cluster2 patients still had significantly worse OS (*p* = 0.045, HR: 1.842 with 95% CI (1.012, 3.355)) and RFS (*p* = 0.041, HR: 1.657 with 95% CI (1.20, 2.692)).

### 2.4. Identification of Genomic Key Features in the HCC Subtypes

**Figure 7:**
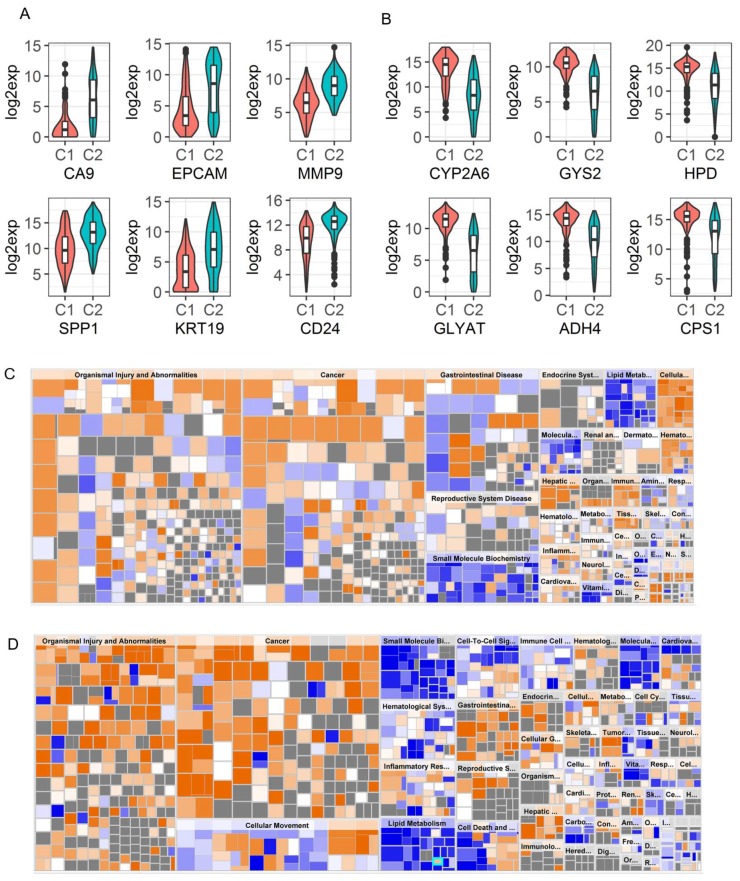
Top differentially expressed genes between the two NMF clusters and Pathway analysis based on the DEGs for TCGA and GSE14520, respectively. (**A**) and (**B**). violin plots to show top up-regulated stemness related genes and top down-regulated metabolic genes, respectively, in Cluster2 (C2) compared to Cluster1 (C1) patients using TCGA data. (**C**) and (**D**). Heatmaps to show the diseases and functions that were affected in Cluster2 compared to Cluster1 based on IPA analysis using the differentially expressed genes between the 2 NMF clusters for TCGA and GSE14520 data, respectively. Orange squares stand for pathways involved in specific diseases and/or functions that were activated and blue squares stand for those that were down-regulated in Cluster2 compared to Cluster1.

In order to understand why Cluster2 patients had significantly worse survival than Cluster1 patients, we identified DEGs in the two Clusters for TCGA and GSE14520 separately. A total of 2332 (Table S6) and 826 (Table S7) genes were found to be differentially expressed in the two data sets by using a fold change cutoff of 2 and 1.5 in TCGA and GSE14520, respectively, due to the fact that relatively fewer genes were differentially expressed in GSE14520 (*p* < 0.00001). Similar to the findings of Woo et al. [17], we found that CA9 was the most upregulated gene in Cluster2 of TCGA data with more than a 20-fold difference (Figure 7A). As a marker of hypoxia, overexpression of CA9 is a poor prognostic marker in HCC, and its expression is associated with stemness-related phenotypes in HCC [17,18]. Our results also showed that increased CA9 expression was significantly associated with a poor prognosis of OS (*p* = 0.0002, HR: 1.085 with 95% CI (1.04, 1.132)). In addition, stemness-related genes such as KRT19, EPCAM, CD24, PROM1, and MMP9 were found to be highly expressed in aggressive Cluster2 tumors compared to Cluster1 as reported [17] (Figure 7A and Table S6). In GSE14520, SOX4, SOX9, MMP9, and CD24 were found to be the top DEGs comparing Cluster2 to Cluster1 (Table S7). CA9 and PROM1 were also significantly overexpressed in Cluster2 even though these are not the most overexpressed genes (Table S7). Notably, SPP1 is one of the top upregulated genes in Cluster2 patients in both data sets (Figure 7A, Tables S6 and S7), which was reported to enhance the stemness of HCC cells [19] and to be involved in PD-L1-mediated immune escape of HCC [20]. The characteristics of Cluster 2 identified by us is very similar to those of iCl1/C1 cluster, as reported by Woo et al. [17]. Collectively, these results indicate that the Cluster2 tumors might be associated with stemness traits, which contribute to the aggressiveness of the tumor.

In contrast, top down-regulated genes in Cluster2 (compared to Cluster1) were involved in metabolic pathways. The top down-regulated genes were CYP family (2A6, 3A4, 8B1, 2A7, 1A2, 7A1), GLYAT, GYS2, ADH4, HSD11B1, HPD, PCK1, ADH1B, CPS1, ABCB11 et al. in TCGA (Figure 7B, and Table S6). Similarly, the top down-regulated genes are SLC10A1, HPD, CPS1, GNMT, SDS, PCK1, ALDOB, ADH1B, and G6PC etc. in GSE14520 (Table S7), which show more severe metabolic dysregulation in Cluster2 than in Cluster1. This could also contribute to a worse prognosis of Cluster2 patients.

To understand which pathways were enriched or dysregulated in Cluster2, a network analysis based on the DEGs between the two clusters were performed for each data using the Ingenuity Pathway Analysis (IPA). This analysis showed that Cluster2 patients had increased risk for organismal injury and abnormalities, cancer, and metabolic disease (Figure 7C,D) in both data sets. In more detail, Cluster2 patients had significantly activated pathways involved in cancer, organismal injury, abnormalities, gastrointestinal disease, and hepatic system disease, and had increased cell proliferation and cellular movement, but had decreased activation of pathways involved in fatty acid metabolism (lipid metabolism and small molecule biochemistry), xenobiotic metabolism, molecular transport, and cell death (*p* < 1 × 10^−12^). In addition, pathway enrichment analysis (GSEA) based on KEGG pathways showed that Cluster2 tumors had decreased activity in multiple metabolism-related pathways (15 commonly dysregulated metabolic pathways between the two Cluster2s from TCGA and GSE14520 were detected), but had increased ribosomal biogenesis, DNA replication, and cell cycle progression (Figure S4A,B). This finding is similar to the results of the HCC group identified by the low expression of ALB and APOB in the liver cancer TCGA landmark study, where this group of patients had increased ribosomal biogenesis and DNA replication [13]. However, these two genes were not differentially expressed between the two clusters in our analysis. Taken together, the results indicate that decreased energy metabolism, reduced cell death and apoptosis, and increased cellular movement and proliferation might be the underlying molecular mechanism that contributes to the worse survival outcomes of Cluster2 patients.

### 2.5. Immunologic Microenvironment in HCC Subtypes

**Figure 8:**
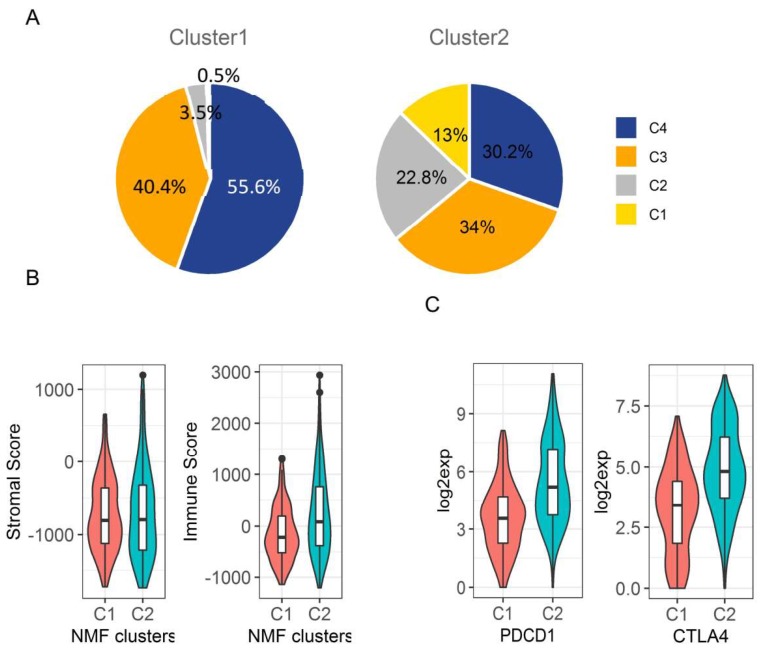
Comparison of the tumor microenvironment between the two NMF clusters using TCGA data. (**A**). Pie charts to show that Cluster2 had significantly higher proportion of patients belonging to immune subtypes 1 and 2 that were associated with worse survival, but had a lower proportion of patients belonging to immune subtypes 3 and 4 that had better survival (*p* < 0.0001). (**B**). Cluster2 tumors had significantly higher immune cell infiltration as measured by the immune score than Cluster1 tumors (*p* < 0.0001), but not the level of stromal cells as measured by the stromal score (*p* > 0.05). (**C**). Cluster2 tumors had significantly higher PD1 (PDCD1) and CTLA-4 expression than Cluster1 tumors (*p* < 0.0001). **Note**: C1–C4 in (**A**) indicates immune subtypes, while C1 and C2 in (**B**) and (**C**) indicates Cluster1 and Cluster2.

Several immune therapies have been applied to HCC patients with advanced disease, such as PD1 and CTLA-4 inhibitors. However, the responses of patients to these therapies are determined by the balance between the antigenicity of the tumor and the microenvironment of cancer tissues [21]. Immune infiltrates in tumour stroma is an important factor that could affect cancer patient survival. Six types of immune infiltrates were identified in human tumours that correspond from tumour-promoting to tumour-suppressive factors, respectively [22,23]. They are C1 (wound healing), C2 (INF-r dominant), C3 (inflammatory), C4 (lymphocyte depleted), C5 (immunologically quiet), and C6 (TGF-beta dominant), where C1, C2, and C6 are associated with a worse prognosis [22]. The majority of HCC patients in the liver cancer TCGA data belonged to C3 and C4 immune subtypes (~80%), and only one patient belonged to C6. There was no C5 immune subtype in liver cancer TCGA data. Interestingly, we found that Cluster2 samples had a significantly increased proportion of C1 and C2 immune subtypes that were associated with a poor prognosis, but had decreased C3 and C4 immune subtypes where C3 corresponds to tumour suppression and was associated with better survival (*p* < 0.0001) (Figure 8A and Figure S5). The stromal and immune cells that form the tumor microenvironment serve a key role in the aggressiveness of tumors. Immune scores based on the ESTIMATE algorithm have been used to measure the level of immune infiltrates in cancer tumors, and the stromal scores to measure the level of stromal cells in the tumor microenvironment [24]. We further compared the immune score and stromal score between those two clusters and found Cluster2 had a significantly increased immune infiltration score compared to Cluster1 (254 ± 62.31 vs. -152.4 ± 34.46, *p* < 0.0001), but there is no significant difference on stromal scores between the two clusters (Figure 8B). In addition, the immune-therapeutic targets PD-1 (PDCD1: programmed cell death protein 1) and CTLA-4 (cytotoxic T-lymphocyte associated protein 4) were significantly upregulated in Cluster2 in TCGA data by more than a 3-fold change (Figure 8C and Table S6). In summary, higher immune infiltration in Cluster2 tumors may further contribute to a poor prognosis but may provide opportunity for Cluster2 patients to be treated with immunotherapy, particularly anti-PD-1 and CTLA-4 inhibitors [25].

## 3. Discussion

As a hallmark of cancer cells, cancer cell metabolism has attracted enormous interest along with the parallel explosion of genomic, transcriptomic, proteomic, and epigenetic profiling of tumors. Based on the rapidly evolving insights on tumor metabolism, Pavlova and Thompson has grouped the emerging metabolism alterations into six hallmarks, among which is the deregulated uptake of glucose and amino acids [26]. In the present study, by integrative transcriptomic analysis of genes within the glucose metabolic pathway, we identified consistently deregulated glucose metabolic genes and interrogated their association with patient tumor stage and outcomes. These findings represent an indispensable step toward exploiting treatment options targeting cancer cell metabolism. In summary, our results showed that more than 30% of glucose metabolic genes (58/179) were consistently deregulated in HCC tumors with a predominant down-regulation (>70%), which is similar to observations previously reported by Nwosu et al. [6]. Although most of the genes were predominantly down-regulated, those involved in the rate limiting steps of glycolysis were mainly up-regulated. This is in line with the well-known metabolic dysregulations in cancer cells hallmarked with accelerated aerobic glycolysis [9]. However, we observed a mixture of both up-regulated and down-regulated genes in most of the metabolic pathways. For example, although accelerated glycolysis is a hallmark of cancer cells, genes involved in this process are predominantly up-regulated. HK3, ALDOB, ENO3, PFKFB1, and PFKFB3 are down-regulated, as are the deregulated glucose transporters (GLUT2, GLUT3 and GLUT9) and the lactate transporters (MCT3 and MCT8). The genes involved in the TCA cycle, glycogen metabolism, and gluconeogenesis are mainly down-regulated. Although it is not clear why HCC tumor cells require down-regulation of those genes, their suppressed expression seem to be beneficial for cancer cell growth, since the down-regulation of many of those genes was associated with worse survival of HCC patients.

There are 14 glucose transporter isoforms (GLUT) encoded by different genes and with different affinities for glucose in humans that facilitate the transport of glucose across the plasma membrane [27]. GLUT1, GLUT2, GLUT3, and GLUT4 are the members that have been commonly studied, and they have been reported to be overexpressed in multiple cancer types, particularly GLUT1 [27,28]. Our study showed that GLUT2, GLUT3, and GLUT9 were significantly down-regulated, and down-regulation of GLUT2 was associated with worse survival. While GLUT5 was consistently upregulated in HCC, the expression of this gene was not an independent predictor for survival, even though it was associated with OS with univariate analysis. A previous study showed that GLUT2 could be a novel prognostic factor for HCC with higher expression than other GLUT family members and is associated with a poor prognosis. This is consistent with our finding that GLUT2 was associated with a higher tumor stage and worse survival, but their study did not show that GLUT2 was down-regulated in tumors [29]. As mentioned above, it is not clear why tumors choose to survive with down-regulation of those transporters. In HCC, GLUT5 might be the major transporter to facilitate the accelerated uptake of glucose. However, the specific role of each isoform in a specific cancer type still needs to be further studied. The first committed step in glucose metabolism is catalyzed by hexokinase, and previous studies showed that cancer cells express a high level of HK2 to increase glucose uptake in lung cancer and breast cancer [30,31]. However, our study found that HK2 was not differentially expressed in HCC tumors. Instead we found that isoform HK3 was consistently decreased while HKDC1 was consistently increased in HCC cells. HKDC1 is a newly identified isoform of hexokinase and was found to contribute to liver metabolism and associate with a poor prognosis of HCC [27,32,33,34]. It is possible that HKDC1 is the predominant form in HCC, but this needs to be further confirmed. The second committed rate-limiting step in glycolysis is the conversion of G6P to F6P catalyzed by PFK1 (phosphofructokinase-1). Our results showed that the platelet isoform of PFK1 gene (PFKP) was significantly increased in HCC, which is also an independent predictor for OS, but the other two isoforms PFKM and PFKL were not. PFKP has been reported to contribute to metabolic reprogramming and maintain cell proliferation in clear cell renal carcinoma [35], and promote tumorigenesis in glioblastoma [36], but has not been well studied in HCC. It could be a potential therapeutic target in HCC and worth further functional study. PFK1 is allosterically activated by fructose 2, 6-biphosphate (F2, 6BP), which is generated from F6P by PFKFB. There are four PFKFB isoforms (PFKFB1-4), among them PFKFB1 and PFKFB3 were found to be consistently down-regulated in HCC, while PFKFB4 was consistently upregulated. These PFKFB isoforms were also significantly associated with patient OS, and PFKFB1 was associated with an advanced tumor stage as well. PFKFB1 has not been well studied and not found to be overexpressed in cancer cells [37]. In contrast to our finding, PFKFB3 was reported to be constitutively overexpressed in different cancer cell lines, several human leukemias, and multiple solid tumors such as ovarian cancer, thyroid cancer, breast cancer, gastric tumors, and pancreatic cancer [37,38,39]. Its expression was found to be associated with lymph node metastasis and the TNM stage [39]. The upregulation of PFKFB4 was reported in multiple cancer types and was associated with the aggressiveness of tumors such as in bladder cancer, breast cancer, lung cancer, and ovarian cancer [40,41,42,43]. This is similar to our finding that upregulation of PFKFB4 is associated with aggressiveness of HCC. The last committed rate-limiting step is the conversion of phosphoenolpyruvate (PEP) to pyruvate catalyzed by pyruvate kinase 2 (PKM2). PKM2 was consistently up-regulated in HCC and it was also an independent predictor for OS and associated with a higher tumor stage. PKM2 has been reported to be universally expressed in cancer and contribute to maintain a metabolic program in cancer cells for tumor growth, even though its role varies in different cancer types [27,44]. In addition to the upregulated expression of genes involved in rate-limiting steps, GPI, ALDOA, GAPDH, BPGM, and ENO1 in the glycolysis pathway were also significantly up-regulated and correlated with OS. ALDOA [45,46,47] and ENO1 were both found to be involved in tumorigenesis in different cancer types [48,49,50,51,52]. BPGM was reported to control serine flux via 3-phosphoglycerate [53], which may benefit cancer cell survival. However, its role in cancer is not clear. Besides the upregulated genes in the glycolysis pathway, G6PD and TKT in the pentose phosphate pathway were also consistently upregulated, which predicted worse survival. This is consistent with previous reported functions of these two genes associated with aggressiveness of the disease and drug resistance in other cancer types [54,55,56,57,58]. Most of the genes involved in glycogen metabolism such as GYS2, UGP2, and AGL had down-regulated expression when compared to normal tissues. Our results showed that expression of GYS2 was significantly upregulated in HCC patients with Non-Alcoholic Fatty Liver Disease (NAFLD) by more than an 8-fold change compared to patients without any primary risk factors (data not shown), which indicates a possible role of GYS2 in NAFLD and is worth further study. In conclusion, the deregulated genes that are also associated with a poor prognosis, especially those with upregulated expression in HCC, could be potential prognostic markers and/or therapeutic targets.

Remarkably, unsupervised classification of the deregulated glucose metabolic genes that were also associated with worse OS in two separate data sets identified two distinct prognostic subtypes of HCC patients (Cluster1 and Cluster2). One subtype of patients (Cluster2) had significantly worse survival including OS, RFS, DSS, PFI, and DFI than the patients in Cluster1. In the multivariate Cox proportional hazard regression model after controlling for age, gender, stage, and other clinical factors, the cluster was still an independent predictor for OS, DSS, and RFS. By comparing the gene expression profiling between the two subtypes, we found that the aggressive Cluster2 tumors had significantly upregulated expression of stemness-related genes such as CA9, EpCAM, MMP9, KRT19, CD24, and PROM1 (CD133). These are well-known markers of stemness traits and are associated with poorer prognostic outcomes in HCC [18,59,60,61,62]. Based on integrative analysis of genomic and epigenomic data in liver cancer, Woo et al. also identified one aggressive cluster of the HCC subtype (iCl1) with the previously mentioned stemness-related genes being on top of the upregulated genes [17], which is similar to our finding. Therefore, we suggest that our identified Cluster2 may share similar functional features to iCl1, such as the stemness or aggressiveness of tumors. Furthermore, we found that Cluster2 tumors had significantly increased immune infiltration compared to Cluster1 tumors. Additionally, more tumors in Cluster2 belonged to immune infiltrate subtypes with a worse prognosis, which might also contribute to a worse prognosis of Cluster2 patients since immune infiltration generally associates with poor prognosis. Notably, Cluster2 tumors exhibited significantly upregulated expression of PDCD1 (PD-1) and CTLA-4, which suggests those patients may be benefited from immuno-therapy, particularly with anti-PD-1 and CTLA-4 inhibitors. While aggressiveness or stemness-related genes were upregulated in Cluster2, genes involved in metabolic pathways were significantly down-regulated. As a consequence, pathway analysis showed that Cluster2 tumors had significantly increased activation of pathways involved in cancer, organismal injury and abnormalities, cell proliferation, ribosome biosynthesis, and DNA replication. However, Cluster2 tumors had decreased activation of pathways involved in cell death and apoptosis, fatty acid metabolism, amino acid metabolism, peroxisome metabolism, propanoate metabolism, drug metabolism, and other metabolic pathways. System biology analysis by Bidkhori et al. based on TCGA data also identified a subtype of HCC patients (iHCC3) with worse survival [63], which had similar properties to our Cluster2 patients since both exhibited activation of pathways associated with tumor aggressiveness and down-regulation of multiple common metabolic pathways. Therefore, the deregulated gene signature in cluster 2 could be prognostic markers and therapeutic targets for highly aggressive HCCs.

## 4. Materials and Methods

### 4.1. Gene List

The dysregulation of glucose metabolic pathway in tumor cells includes genes involved in glycolysis, citrate acid (TCA) cycle, and pentose phosphate pathway, which breaks down glucose to provide metabolites for necessary energy production, as well as gluconeogenesis, glycogenolysis, and glycogenesis pathways that can balance the glucose level in blood, and genes regulating those processes. Most of the glucose metabolic genes used in this study were initially downloaded from the KEGG pathway database (http://www.genome.ad.jp/kegg/pathway.html) using an R package (KEGGREST), including has00010 Glycolysis/Gluconeogenesis, has00020 Citrate cycle (TCA cycle), has00030 Pentose phosphate pathway, and has00050 glycogen metabolism (including both glycolysis and glycogen metabolism pathway genes). We combined the KEGG genes, and then removed the overlapping genes. In addition, glucose and lactate transporter genes, glucose metabolism and glycogen metabolism regulating genes were also collected and included, which resulted in 179 glucose metabolic genes being used for this study (Table S4).

### 4.2. Datasets and Gene Expression

To compare expression of genes within the glucose metabolic pathway between patient tumor and normal tissues, TCGA liver cancer RNA-seq (LIHC) data, and liver cancer microarray gene expression datasets GSE14520, GSE87630, GSE76427, and GSE39791 were used in this study. The platform and number of samples used from each data set was listed in Table S1. LIHC TCGA data, including RNA-Seq (RNA SeqV2 RSEM), clinical data, and immune subtypes were downloaded from the xena browser (https://xenabrowser.net/datapages/). The tumour samples in TCGA are surgical resection samples obtained from primary tumours that have received no prior neoadjuvant treatment. All the microarray data sets including clinical data of GSE14520 are publicly available at the Gene Expression Omnibus (GEO). To compare gene expression between tumors and adjacent normal tissues, linear mixed effects models were used for datasets TCGA, GSE76474, GSE39791, and GSE14520 to take account of correlation among samples from the same patient. Since GSE14520 contains two sets of data from different platforms (GPL571 and GPL3921), a batch effect was controlled in the analysis. ANOVA (Analysis of Variance) was used to compare gene expression of GSE87630 since it includes independent samples. If multiple probes corresponded to the same gene, the final expression of the gene was determined by the arithmetic mean of multiple probes before comparison. All genes with a *p*-value < 1/180 = 0.0056 (controlling 1 false positive among the tested genes) and a fold-change ≥1.5 were claimed to be significantly differentially expressed for each data set. The genes that were consistently differentially expressed (DEGs) in at least two of the five data sets were further considered in the analyses.

### 4.3. Survival Analysis

The association between the expression of the DEGs and patient clinical outcomes including OS (overall survival), PFI (progression free interval), DSS (disease specific survival), and DFI (disease free interval) for TCGA, OS and RFS (recurrence free survival) for GSE14520 were tested with univariate or multivariate Cox-proportional hazard regression models (age, gender, and tumor stage was controlled). Forest plots were used to display the results. A *p*-value < 0.05 was considered significant in order to be consistent with the forest plots. For testing the association between gene expression and other clinical parameters such as tumor stage, ANOVA or the Spearman Correlation were applied.

### 4.4. Clustering Analysis

To investigate whether genes that were differentially expressed in at least two data sets and were also associated with OS could predict prognostic patient subgroups, non-negative factorization (NMF) clustering with a standard “Brunet” method was performed to identify stable clusters. The number of clusters k was set to 2 to 5 but k = 2 was chosen based on the observed consensus map and cophenetic correlation between clusters. R package NMF was used for this analysis.

### 4.5. Comparison of NMF Clusters

To investigate the difference between the two identified NMF clusters for TCGA and GSE14520 data, a Log-rank test was used to compare survival outcomes such as OS, DSS, PFI, DFI, and RFS. In addition, multivariate Cox-proportional hazard regression models were used to test whether the NMF clusters are still an independent prognostic predictor after controlling for patient clinical characteristics such as age, gender, tumor stage, and tumor size. Two-sample t-tests were used to compare continuous variables such as age. Chi-square analysis was used to compare categorical variables such as gender, tumor size, and tumor stage (dichotomized into early (I, II) and late stage (III,IV)), AFP, and ALT level. Gene expression was compared between the two clusters with moderated t-tests. Controlling the number of the false positive method was used to control for Type I error. Fold change (FC) of 2 and 1.5 were used as the cutoff for TCGA data and GSE14250, respectively, to choose genes for following pathway analysis, considering the fact that the microarray data, in general, cannot detect as many differentially expressed genes as RNA-sequencing.

### 4.6. Gene Set Enrichment Analysis between NMF Clusters

To understand the mechanism why the NMF clusters had different survival outcomes, the differentially expressed genes between the two clusters were used to conduct pathway analysis. Core analysis of Ingenuity Pathway Analysis (IPA) was used to investigate the enriched or dysregulated pathways (www.ingenuity.com). In addition, KEGG pathways were used to investigate the metabolic pathway differences between the two clusters with Gene Set Enrichment Analysis (GSEA).

### 4.7. Tumour Microenvironment and Immune Subtype Analyses

The ESTIMATE immune score and stromal score were used to analyze the infiltration levels of immune cells and stromal cells in different tumours [24]. This analysis was based on the interpretation of gene expression profiles retrieved from TCGA expression data (http://bioinformatics.mdanderson.org/estimate/) [24]. The difference of the scores between the clusters were tested with ANOVA. Six immune subtypes were defined to measure immune infiltrates in the tumour environment [23]. A chi-square test was used to test the association between the two NMF clusters predicted based on the glucose metabolic pathway and immune infiltrate types in the tumour microenvironment.

### 4.8. Statistical Analysis

Statistical analyses were performed as described above for each individual study using SAS9.4 (SAS Institute Inc., Cary, NC, USA). Plots were created using R (R Core Team) with packages ggplot2, pheatmap, corrplot, or survminer as appropriate [64]. We used a controlling number of the false positives method to adjust for multiple comparisons for all the tests except for the survival study to control the familywise error rate at α = 0.05 [65]. In more detail, we controlled for 1 false positive among the total number of tests within each study to set the cutoff for statistical significance. For example, we used *p* = 1/(180) = 0.0056 as cutoff for comparing gene expression within glucose metabolism in the tumour to adjacent normal since there were around 180 genes. For the survival study, we used *p* = 0.05 as the cutoff without adjusting for multiple comparisons in order for the data interpretation to be consistent with the display of the forest plots results.

All the data sets used in this study are publicly available, and additional data supporting the conclusions of this study are included within the article (Tables S1–S7 and Figures S1–S5).

## 5. Conclusions

In this integrative study, we identified consistently dysregulated genes within glucose metabolic pathways, and investigated their prognostic power for patient outcomes in HCC, including OS, RFS, DSS, RFI, and PFI. Most importantly, for the first time, based on the dysregulated genes that were also associated with OS, we identified two distinct molecular HCC subtypes with one subtype having significant worse prognosis, which could provide novel mechanistic and clinical insights for the development of precision diagnosis and therapeutic options for HCC patients.

## Supplementary Materials

The following are available online at https://www.mdpi.com/2072-6694/11/12/2023/s1. Figure S1: Heatmap of the 47 DEGs between primary tumor and normal tissues that were also significantly associated with OS, Figure S2: NMF clustering diagnostic plots for (A) TCGA and (B) GSE14520, Figure S3: Kaplan-Meier survival plots of OS and DSS for TCGA, and OS and RFS for GSE14520 data with Cluster and Stage as independent variables, Figure S4: KEGG pathway analysis of TCGA and GSE14520 based on the DEGs between the two clusters, Figure S5: Kaplan-Meier survival plot of immune subtypes for TCGA data, Table S1: Summary of the data sets used for this study including number of samples and platforms used for each data set, Table S2. Comparison of patient clinical factors between the two NMF clusters for TCGA data, Table S3: The comparison of patient demographics and clinical characteristics between the two NMF clusters of GSE14520, Table S4: Glucose metabolic gene list used for this study, Table S5: List of genes that are consistently differentially expressed in tumors compared to normal in at least two of five data sets (FC ≥ 1.5 and *p* < 0.0001) and are also associated with OS based on univariate survival analysis, Table S6: Differentially expressed genes with ≥2-fold change and *p* < 0.00001 between the two NMF clusters of TCGA data, Table S7: Differentially expressed genes with ≥1.5-fold change and *p* < 0.00001 between the two NMF clusters of data GSE14520.
