# Supplementary material for: Identification of a Subtype of Hepatocellular Carcinoma with Poor Prognosis Based on Expression of Genes within the Glucose Metabolic Pathway
Source: Cancers (Basel). 2019 Dec 14;11(12):2023. doi: 10.3390/cancers11122023 (PMC6966574; doi:10.3390/cancers11122023)
Supplement: Supplementary file 1 [file cancers-11-02023-s001.zip › cancers-655104-suppl-XML/cancers-655104-Figures S1-5 and Tables S1-3.docx]

Supplementary Materials: Identification of A Subtype of Hepatocellular Carcinoma with Poor Prognosis Based on Expression of Genes within the Glucose Metabolic Pathway

Xiaoli Zhang, Jin Li, Kalpana Ghoshal, Soledad Fernandez and Lang Li

**Figure S1.** Heatmap of the 47 differentially expressed genes between primary tumor and normal tissues that were also significantly associated with OS from univariate Cox proportional hazard regression analysis based on TCGA data, including 371 tumor samples and 50 adjacent normal samples. The heatmap indicates two different clusters of tumor samples with one being grouped together with normal samples.


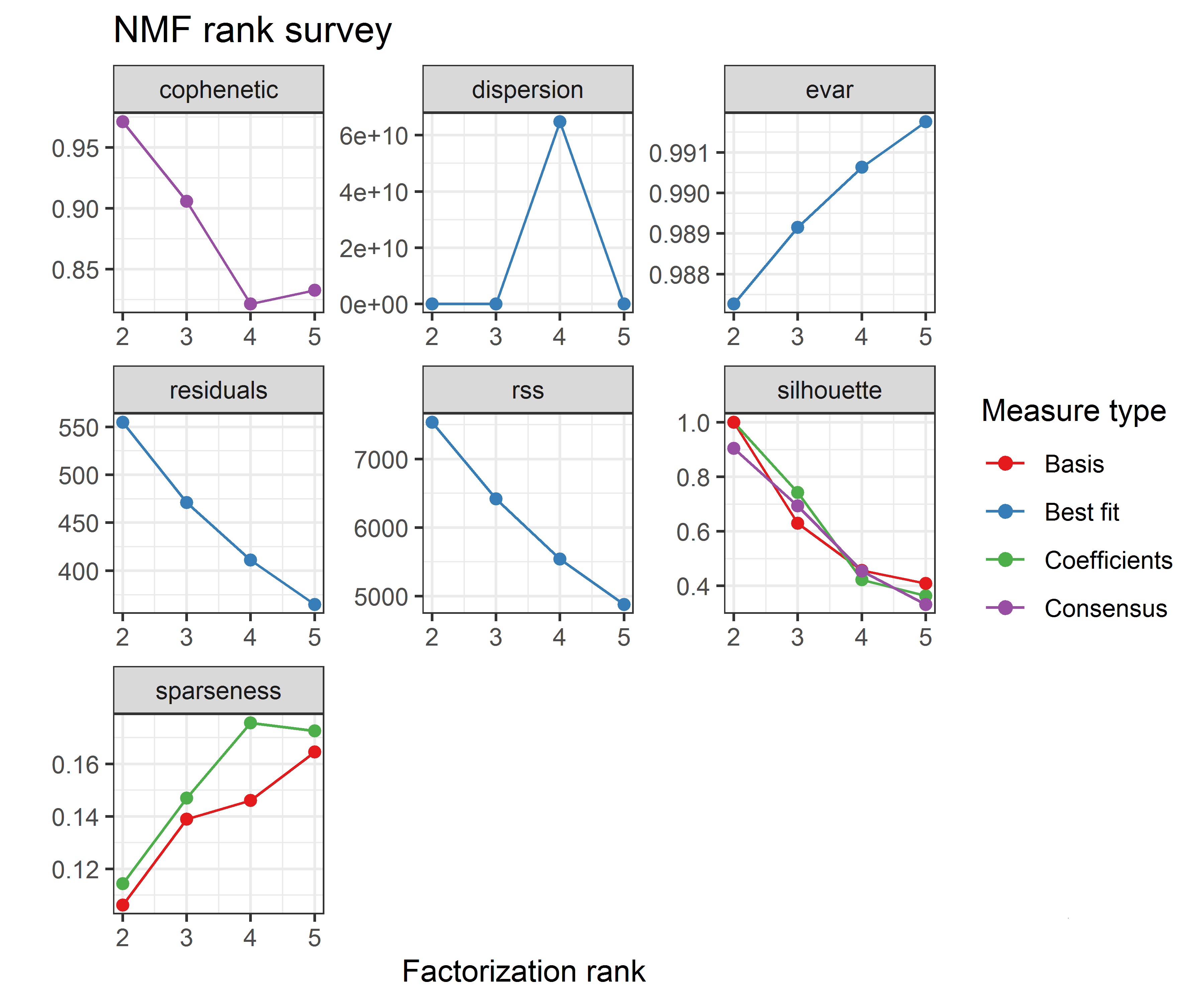

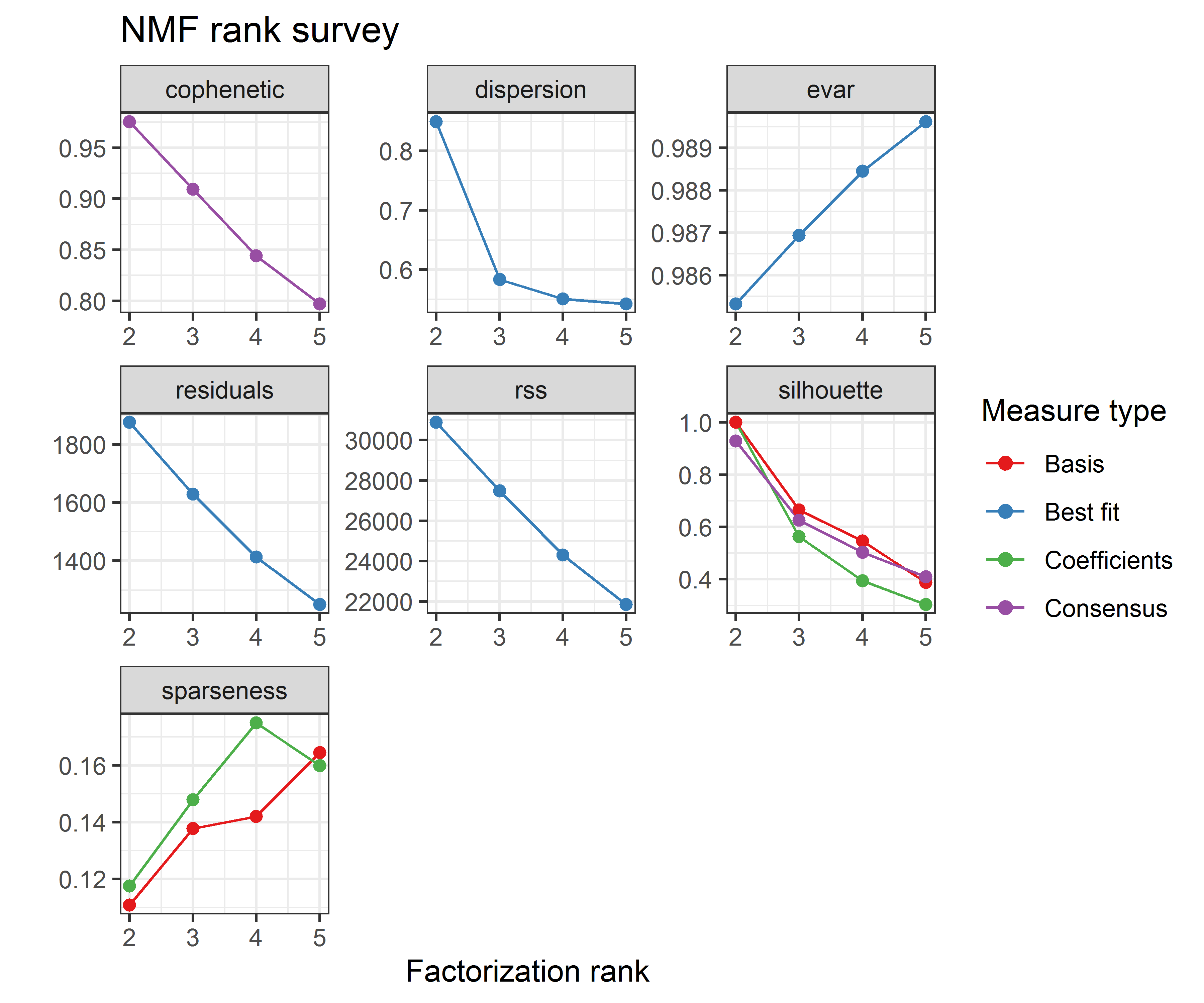


**A**

**B**

**Figure S2.** NMF clustering diagnostic plots for (A) TCGA and (B) GSE14520 showing that K=2 is the optimal choice for clustering based on the cophenetic coefficient.

**A**

**B**


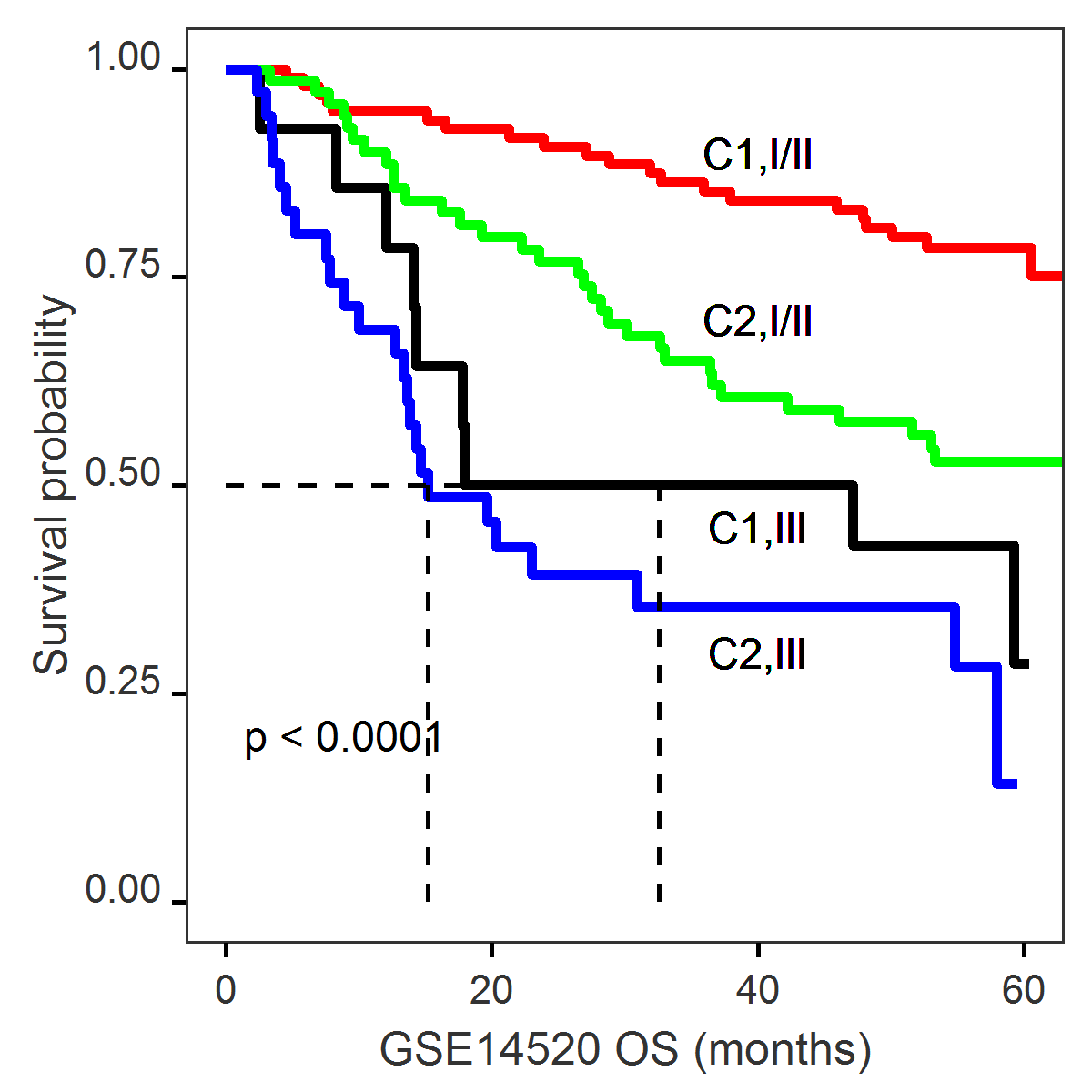

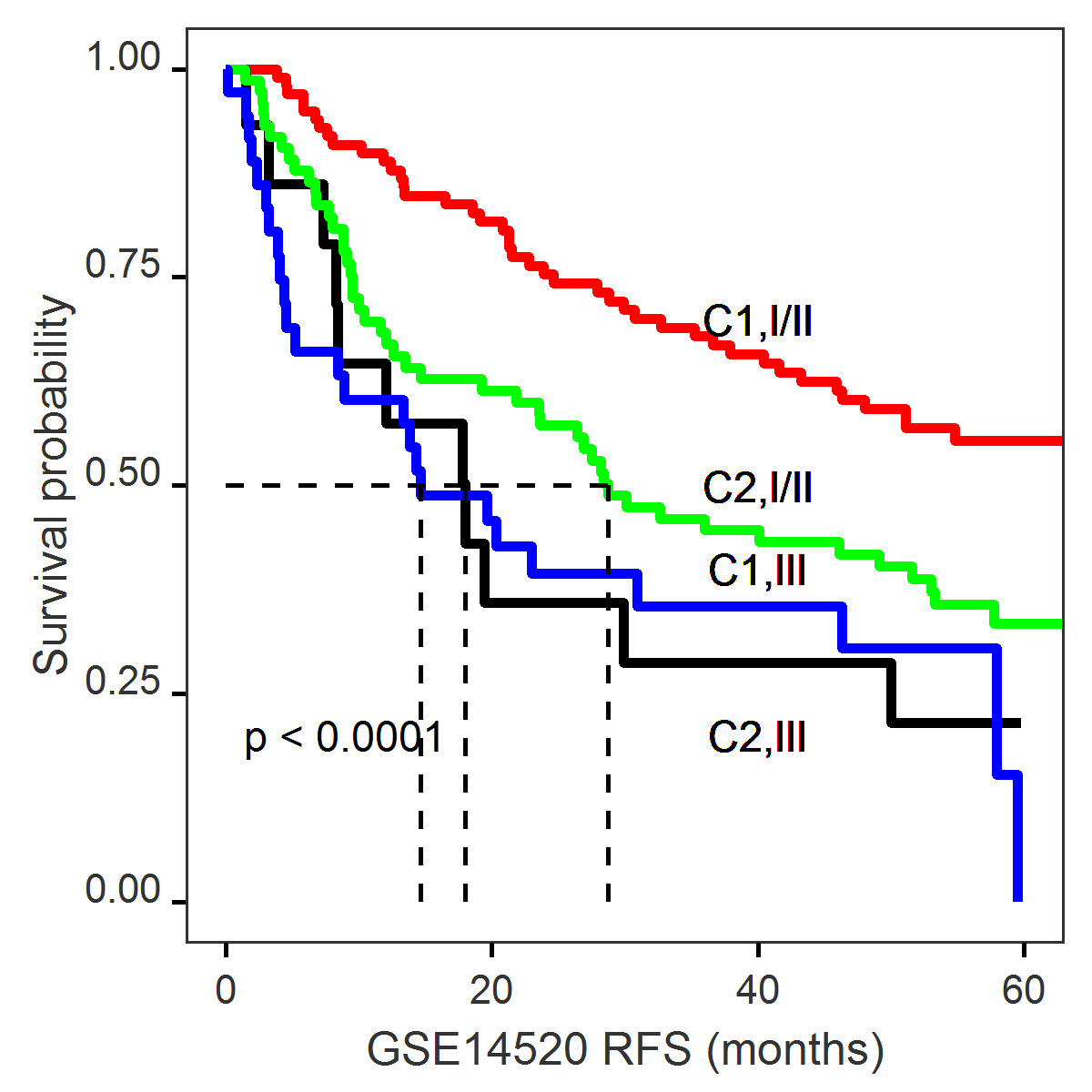

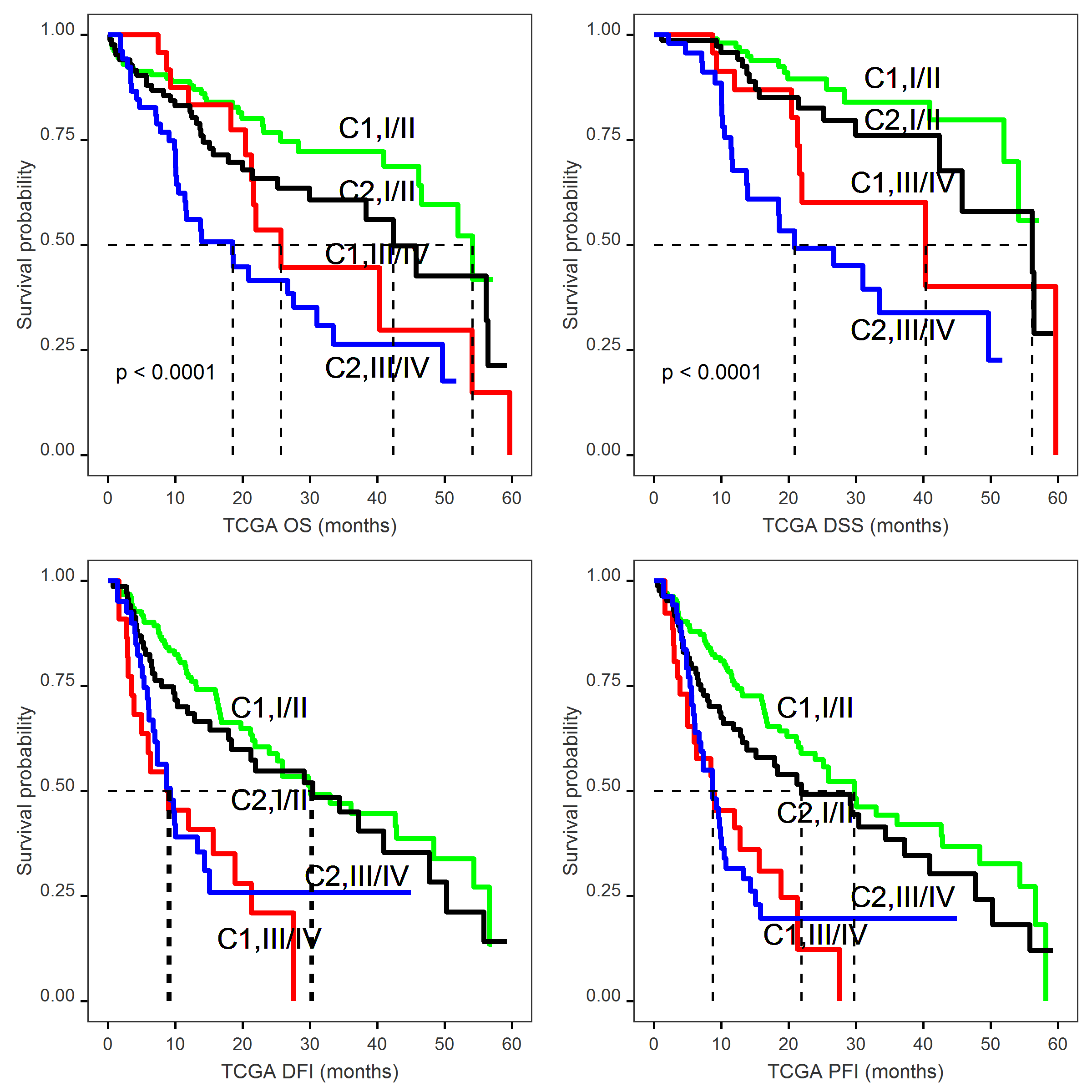


**Figure S3.** Kaplan-Meier survival plots to show that after including tumor stage in the Log-rank test, there was still significant difference between the two NMF clusters on OS and DSS for TCGA, and OS and RFS for GSE14520 data. The difference for OS, RFS and DSS between the two clusters is significant within both early and late stages (*p* < 0.0001), but not for PFI and DFI (*p* > 0.05).


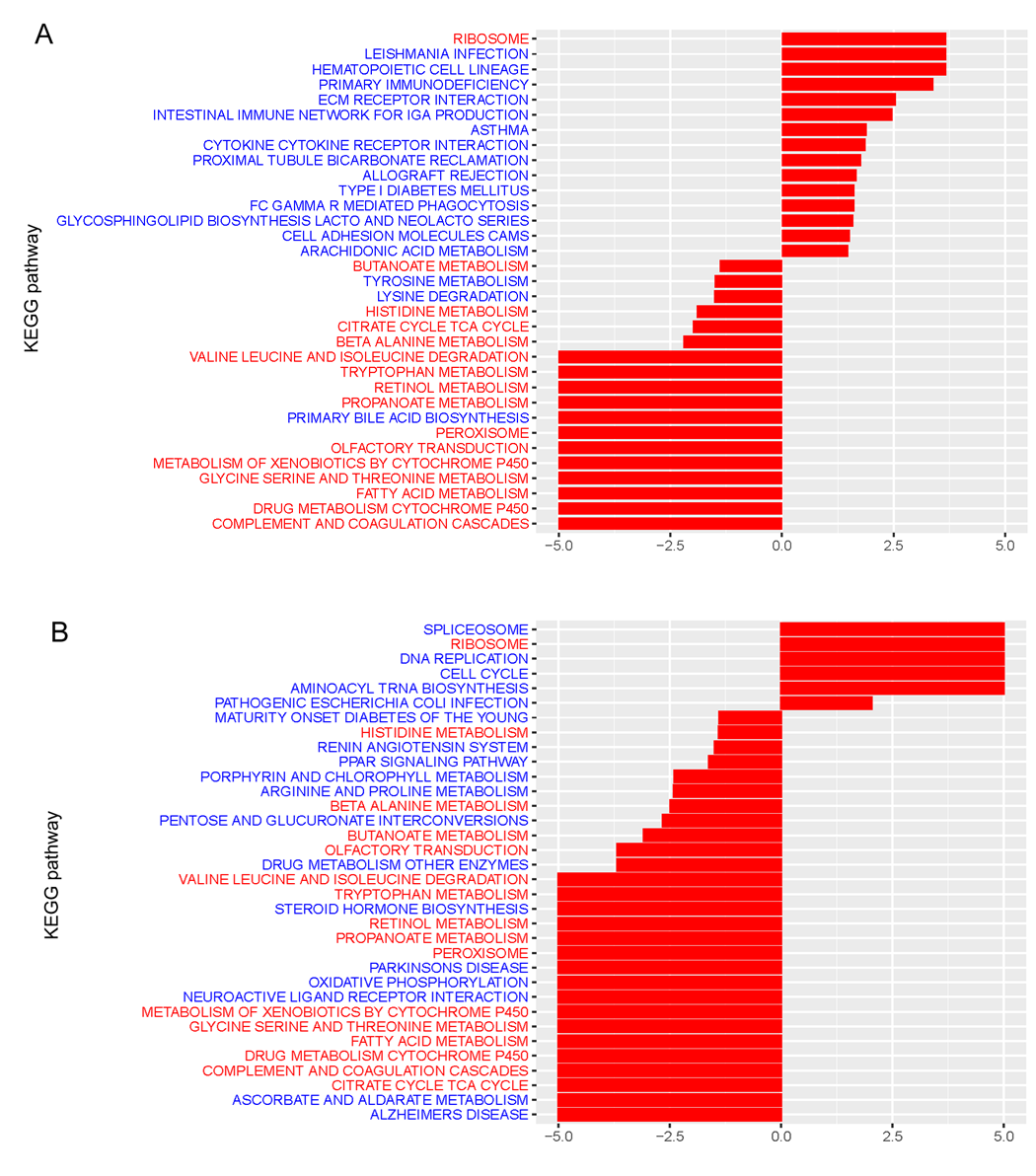


**Figure S4.** Pathway enrichment analysis (GSEA) based on KEGG pathways using the differentially expressed genes comparing cluster 2 to cluster1 showing that Cluster2 tumors had decreased activity in multiple metabolic related pathways, but had increased ribosome biogenesis, DNA replication, and cell cycle progression. Red labels: commonly dysregulated metabolic pathways between the two clusters for both TCGA and GSE14520. Blue labels: the dysregulated pathways specific for each data set. X-axis stands for –log10 (*p*-values) with arbitrary sign to show activated (>0) or down regulated (<0) pathways in Cluster2 compared to Cluster1. (**A**). Pathway analysis for TCGA data. (**B**). Pathway analysis for GSE14520 data.


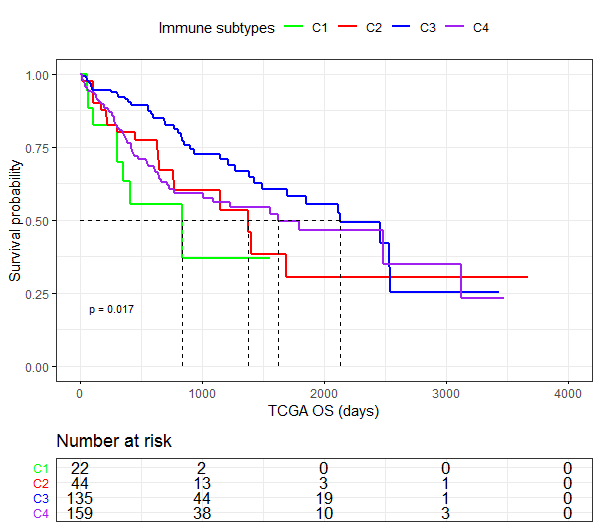


**Figure S5.** Kaplan-Meier survival plot to show survival difference among different immune subtypes using TCGA data. Immune subtype 3 had significantly better survival than other clusters (*p* = 0.017).

**Table S1.** Summary of the data sets used for this study including number of samples and platforms used for each data set.

| **Data Sets** | **Non-Tumor** | **Primary Tumor** | **Paired** | **Platform** |
| --- | --- | --- | --- | --- |
| TCGA | 50 | 371 | yes | RNA-Seq |
| GSE87630 | 30 | 64 | No | GPL6947 Illumina HumanHT-12 V3.0 expression beadchip |
| GSE14520 | 239 | 247 | Yes | GPL571 [HG-U133A_2] Affymetrix Human Genome U133A 2.0 Array,  GPL3921 [HT_HG-U133A] Affymetrix HT Human Genome U133A Array |
| GSE76427 | 52 | 115 | Yes | GPL10558 Illumina HumanHT-12 V4.0 expression beadchip |
| GSE39791 | 72 | 72 | Yes | GPL10558 Illumina HumanHT-12 V4.0 expression beadchip |

**Table S2.** Comparison of patient clinical factors between the two NMF clusters for TCGA data.

| **TCGA**  **Patient Characteristics** | **Levels** | **Cluster1**  **(Normal-Like)**  **( *n*= 203)** | **Cluster2**  **(Aggressive)**  **( *n*= 168)** | ***p*-Value** |
| --- | --- | --- | --- | --- |
| Age (*n* = 370) | Age at initial diagnosis | 61.3±12.2  (*n* = 203) | 57.1 ±14.7 (*n* = 107) | 0.0029 |
| Gender (*n* = 371) | Female | 52 (25.62%) | 69 (41.07%) | 0.0016 |
|  | Male | 151 (74.38%) | 99 (58.93%) |  |
| Race (*n* = 359) | Asian | 82 (41.62%) | 76 (46.91%) | 0.59 |
|  | Black | 10 (5.08%) | 7 (4.32%) |  |
|  | White | 105 (53.30%) | 79 (48.77%) |  |
| Pathologic stage (*n* = 346) | Stage I,II | 157 (83.07%) | 100 (63.29%) | <0.0001 |
|  | Stage III,IV | 32 (16.93%) | 58 (36.71%) |  |
| OS  (*n* = 370) | Censored | 147 (72.41%) | 93 (55.69%) | <0.0001 |
|  | Death | 56 (27.59%) | 74 (44.31%) |  |
|  | Median survival | 87.87 (54.13, 84.73) | 33.50 (25.23,56.17) |  |
| DSS  (*n* = 362) | Censored | 166 (83.0%) | 117(72.22%) | 0.0009 |
|  | Death | 34 (17.0%) | 45 (27.78%) |  |
|  | Median survival | 84.4 (81.87, 104.17) | 56.17 (37.83, ) |  |
| PFI  (*n* = 370) | Censored | 111 (54.68%) | 79 (47.31%) | 0.006 |
|  | Death | 92 (45.32%) | 88 (52.69%) |  |
|  | Median survival | 29.73 (21.3, 42.63) | 13.27 (10.03, 21.23) |  |
| DFI  (*n* = 316) | Censored | 104 (57.46%) | 69 (51.11%) | 0.042 |
|  | Death | 77(42.54%) | 66 (48.89%) |  |
|  | Median survival | 33 (23.97, 54.33) | 18.3 (11.83, 37.23) |  |

**Table S3.** Comparison of patient clinical characteristics between Cluster1 and Cluster2 in GSE14520 data.

| **GSE14520**  **Patient Characteristics** | **Levels** | **Cluster1**  **(Normal-Like)**  **(*n* = 121)** | **Cluster2**  **(Aggressive)**  **(*n* = 121)** | ***p*-Value** |
| --- | --- | --- | --- | --- |
| Age (*n* = 242) | Age at initial diagnosis | 52.29±11.15  (*n* = 121) | 49.39 ±10.46 (*n* = 121) | 0.037 |
| Gender (*n* = 235) | Female | 16 (13.91%) | 14 (12.72%) | 0.95 |
|  | Male | 99 (86.09%) | 96 (87.28%) |  |
| AFP (*n* = 238) | Low | 81 (68.07%) | 47 (39.50%) | <0.0001 |
|  | High | 38(31.93%) | 72 (60.50%) |  |
| ALT (*n* = 242) | Low | 78 (64.6%) | 64 (52.89%) | 0.068 |
|  | High | 43(35.54%) | 57 (47.11%) |  |
| Predicted risk of metastasis (*n* = 242) | Low | 102 (84.30%) | 19 (15.70%) | <0.0001 |
|  | High | 19(15.70%) | 102(84.30%) |  |
| Tumor size  (*n* = 224) | Small | 86 (71.67%) | 67 (55.37%) | 0.0086 |
|  | Large | 34 (28.33%) | 54 (44.63%) |  |
| Pathologic stage (*n* = 230) | Stage I,II | 100 (85.47%) | 74 (65.49%) | 0.0004 |
|  | Stage III,IV | 17 (14.52%) | 39 (34.51%) |  |
| OS  (*n* = 242) | Censored | 87 (71.90%) | 59 (48.76%) | <0.0001 |
|  | Death | 34 (28.10%) | 62 (41.34%) |  |
|  | Median survival  (mons) | Not achieved | 46.1 (28.2, ) |  |
| RFS  (*n* = 242) | Censored | 64 (52.89%) | 42 (34.71%) | 0.0004 |
|  | Death | 57 (47.11%) | 79 (65.29%) |  |
|  | Median survival (mons) | Not achieved | 26.4 (14.3, 36.0) |  |

| 1. 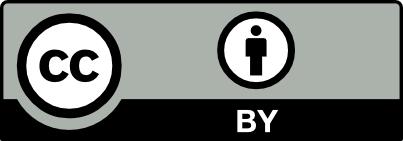 | 1. © 2019 by the authors. Licensee MDPI, Basel, Switzerland. This article is an open access article distributed under the terms and conditions of the Creative Commons Attribution (CC BY) license (http://creativecommons.org/licenses/by/4.0/). |
| --- | --- |
